# Supplementary figures and images for: Machine-learning-based prediction of disability progression in multiple sclerosis: An observational, international, multi-center study
Source: PLOS Digit Health. 2024 Jul 25;3(7):e0000533. doi: 10.1371/journal.pdig.0000533 (PMC11271865; doi:10.1371/journal.pdig.0000533)

ROC-AUC per center - Attention

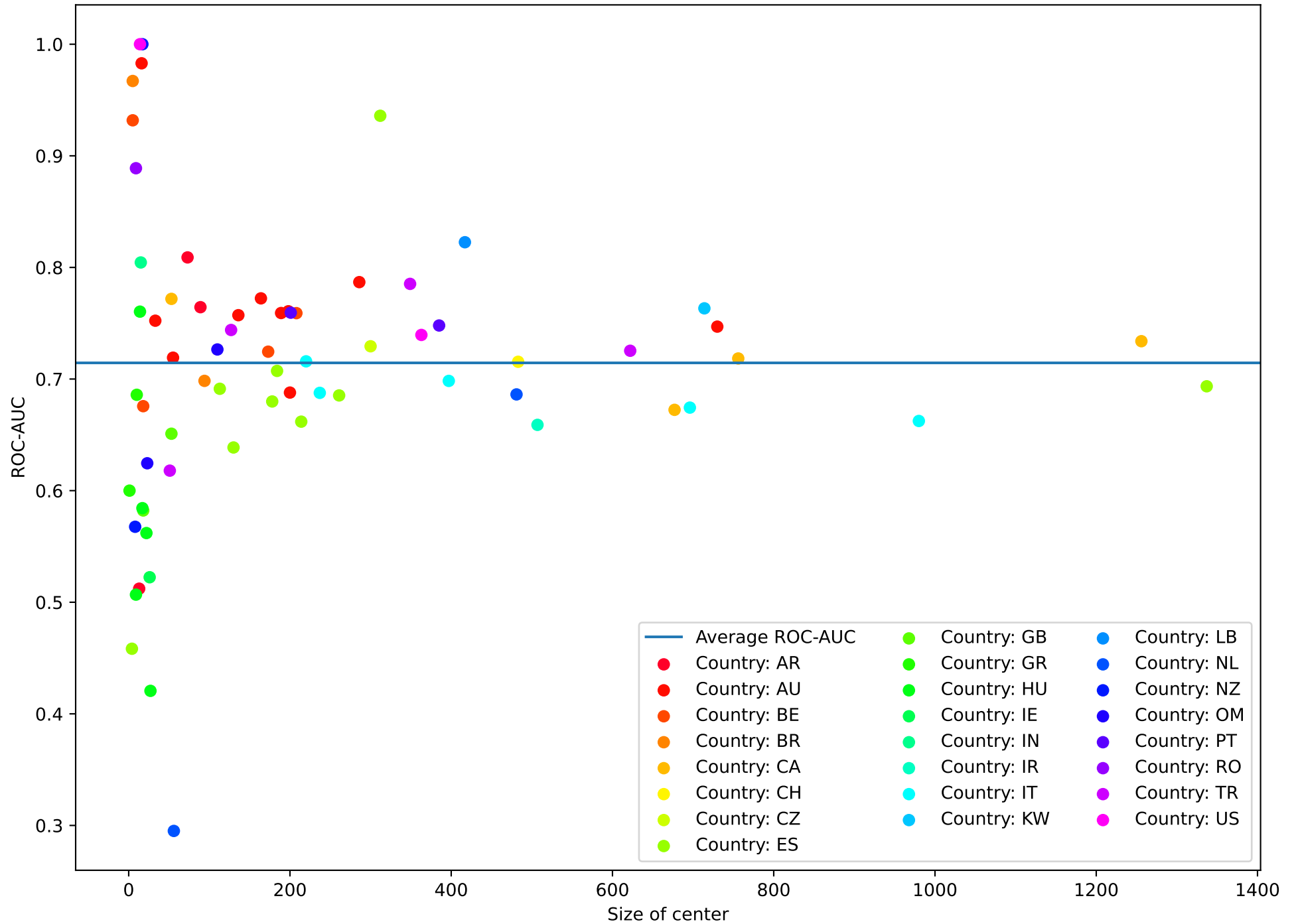

Supplement: S1 Fig — ROC-AUC of individual centers in the test set against the size of the center. As the size of the centers grows, the performance converges to the average ROC-AUC. As the size of centers shrinks, the variability in performance increases, which is statistically expected due to low sample size. Centers with no progression are not plotted (because ROC-AUC is not defined in this case). (PDF) [file pdig.0000533.s001.pdf]

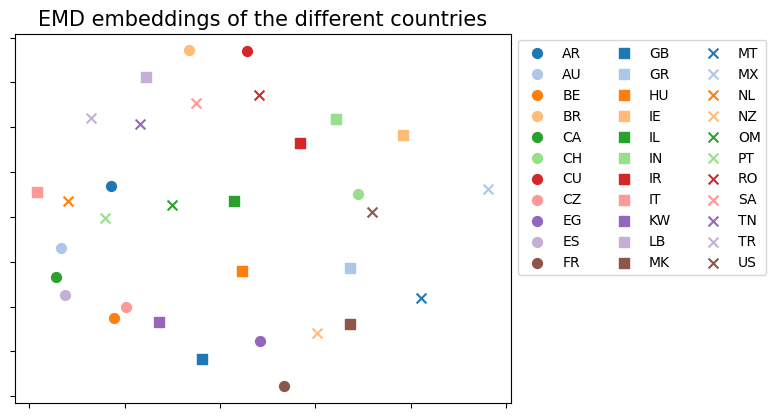

Supplement: S2 Fig — Each country is represented as the set of vectors of static variables for each episode. A distance between countries was computed using earth mover distance. The 2D visualization was obtained by using multidimensional scaling (MDS). (PNG) [file pdig.0000533.s002.png]

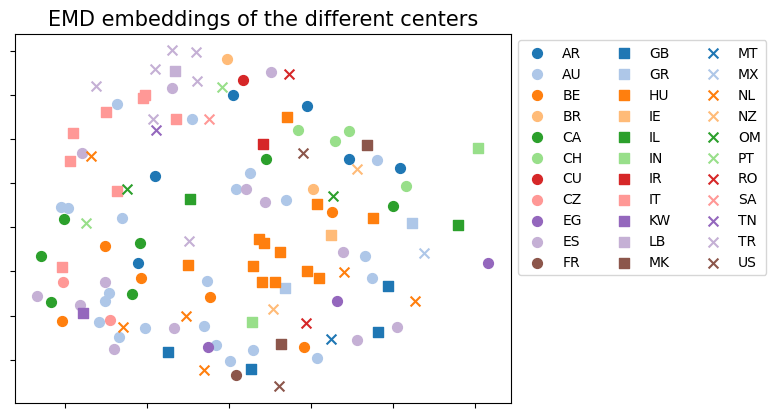

Supplement: S3 Fig — Each center is represented as the set of vectors of static variables for each episode. A distance between centers was computed using earth mover distance. The 2D visualization was obtained by using multidimensional scaling (MDS). We color each center by its country of origin. (PNG) [file pdig.0000533.s003.png]

Comparison of observed incidences and predictions

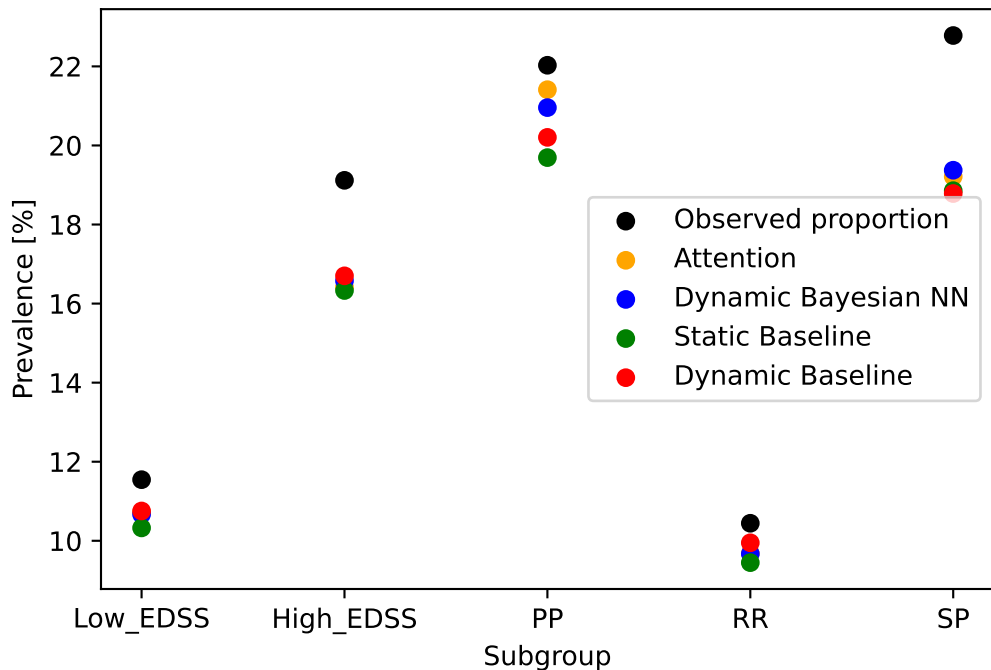

Supplement: S5 Fig — Predicted percentage of worsening per subgroup, for both MS Courses and EDSS larger or smaller than 5.5. Green is the actual prevalence for the age groups on the x-axis, and red and purple are model predictions. This shows the calibration performance for different subgroups. An acceptable discrepancy is observed (of maximum 3 points), and a tendency of the models to underestimate the prevalence of disability progression. (PDF) [file pdig.0000533.s005.pdf]
