# Supplementary material for: Machine-learning-based prediction of disability progression in multiple sclerosis: An observational, international, multi-center study
Source: PLOS Digit Health. 2024 Jul 25;3(7):e0000533. doi: 10.1371/journal.pdig.0000533 (PMC11271865; doi:10.1371/journal.pdig.0000533)
Supplement: S4 Fig — Calibration curves of the different models on the test set (fold (e.g. train-test split) 0). Calibration was performed using Platt scaling [33]. A good calibration was observed for all models. The discrepancy with the ideal line (dotted) in the larger scores regime can be explained by the lowest number of data points in that region, leading to more variance. (PDF) [file pdig.0000533.s004.pdf]

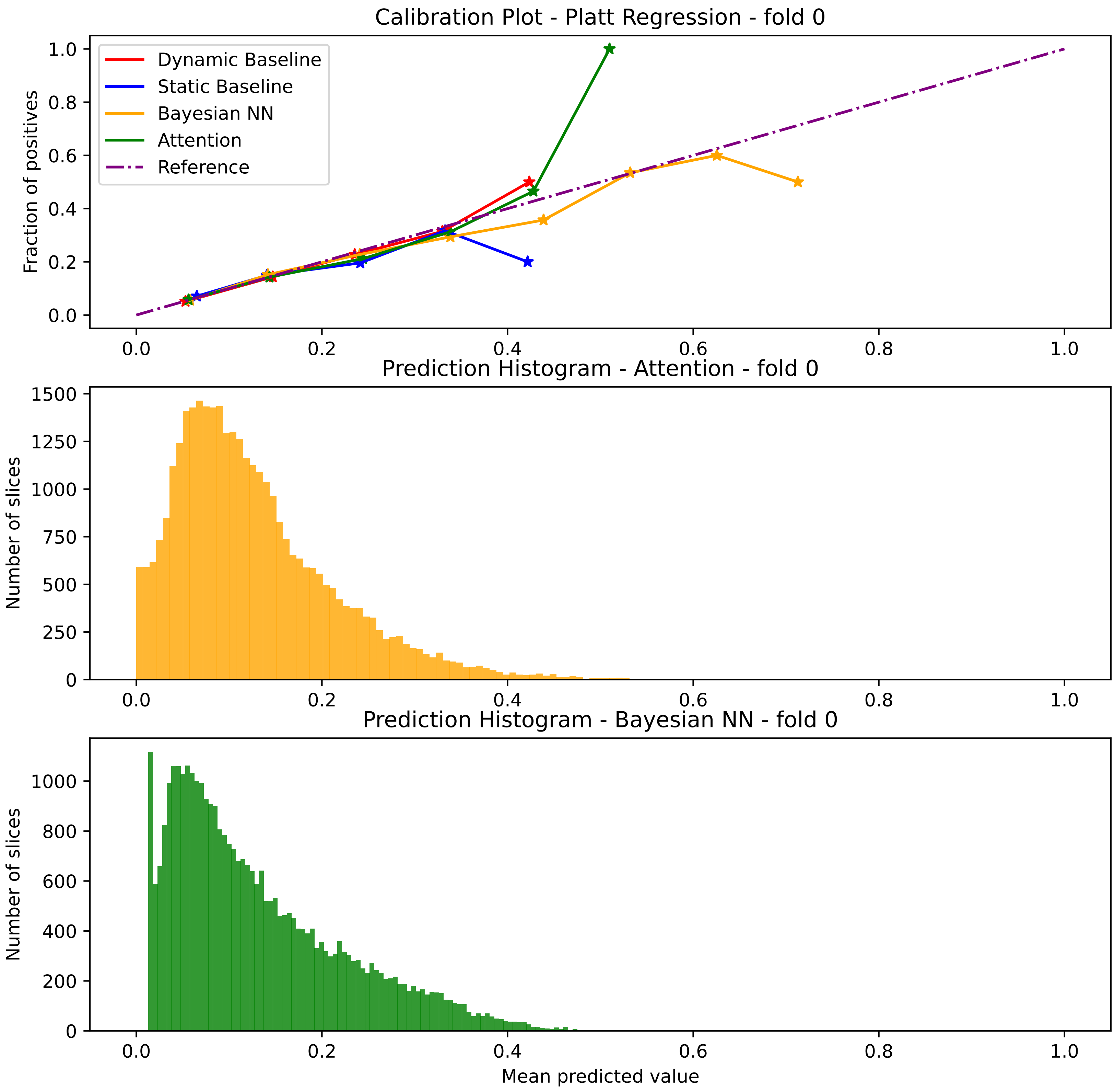

Calibration Plot - Platt Regression - fold 0
Fraction of positives
Dynamic Baseline
Static Baseline
Bayesian NN
Attention
Reference
0.0
0.2
0.4
0.6
0.8
1.0
Prediction Histogram - Attention - fold 0
Number of slices
0
250
500
750
1000
1250
1500
Prediction Histogram - Bayesian NN - fold 0
Number of slices
0
200
400
600
800
1000
Mean predicted value
