# Supplementary material for: Machine-learning-based prediction of disability progression in multiple sclerosis: An observational, international, multi-center study
Source: PLOS Digit Health. 2024 Jul 25;3(7):e0000533. doi: 10.1371/journal.pdig.0000533 (PMC11271865; doi:10.1371/journal.pdig.0000533)
Supplement: S1 Table — Summary statistics of the cohort of interest after patient and sample selection. For all variables the value at the last recorded visit was used. KFS stands for Kurtzke Functional Systems Score, DMT for Disease Modifying Therapy, CIS for Clinically Isolated Syndrome. (PDF) [file pdig.0000533.s006.pdf]

Variable	Cohort 3 EDSS	Cohort 6 EDSS
Patients (% female)	26,246 (71·8)	15,240 (72·0)
Age, Years[a]	42·8 (10·8)	41·9 (10·1)
Age at MS onset, years[a]	31·3 (8·9)	30·6 (8·6)
Disease duration, years[a]	11·6 (8·0)	11·4 (7·3)
Education status, % higher[c]	18·2 (65·1)	16·6 (66·2)
First symptom, none given (%)	13·7	13·0
supratentorial (%)	28·2	30·6
optic pathways (%)	22·6	23·2
brainstem (%)	24·3	25·7
spinal cord (%)	26·4	25·8
MS course	/	/
CIS (%)	0	0
Relapsing-Remitting (%)	83·5	85·7
Primary Progressive (%)	5·0	4·1
Secondary Progressive (%)	11·5	10·2
EDSS[a]	3·0 (2·1)	3·1 (2·0)
Annualized relapse rate[b]	0·82 [0·43, 1·47]	0·86 [0·49, 1·43]
KFS Scores	/	/
pyramidal[b]	2 [1, 3]	2 [1, 3]
cerebellar[b]	0 [0, 2]	0 [0, 2]
brainstem[b]	0 [0, 1]	0 [0, 1]
sensory[b]	1 [0, 2]	1 [0, 2]
sphincteric[b]	0 [0, 1]	0 [0, 1]
visual[b]	0 [0, 1]	0 [0, 1]
cerebral[b]	0 [0, 1]	0 [0, 1]
ambulatory[b]	0 [0, 1]	0 [0, 1]
DMT	/	/
none	23·5	19·0
low-efficacy	51·3	52·0
moderate-efficacy	13·6	14·8
high-efficacy	11·6	14·1
high induction	7·2	7·4

a: mean ± standard deviation
b: median (quartiles)
c: % missing data
