# Supplementary material for: Machine-learning-based prediction of disability progression in multiple sclerosis: An observational, international, multi-center study
Source: PLOS Digit Health. 2024 Jul 25;3(7):e0000533. doi: 10.1371/journal.pdig.0000533 (PMC11271865; doi:10.1371/journal.pdig.0000533)
Supplement: S3 Table — ROC-AUC, AUC-PR, Brier Score and ECE of all models (averages ± standard deviations). Cohort of patients with a least 6 visits with EDSS in the last 3.25 years. (PDF) [file pdig.0000533.s008.pdf]

Model	ROC-AUC	AUC-PR	Brier	ECE
Ensemble	0.71 ± 0.02	0.25 ± 0.03	0.11 ± 0.01	0.08 ± 0.03
Attention	0.7 ± 0.02	0.24 ± 0.02	0.11 ± 0.01	0.04 ± 0.01
RNN	0.7 ± 0.02	0.24 ± 0.03	0.11 ± 0.01	0.05 ± 0.03
Static Bayesian NN	0.68 ± 0.02	0.23 ± 0.02	0.11 ± 0.01	0.05 ± 0.03
Dynamic Bayesian NN	0.7 ± 0.01	0.24 ± 0.02	0.11 ± 0.01	0.08 ± 0.02
Static Baseline	0.67 ± 0.02	0.22 ± 0.02	0.11 ± 0.01	0.05 ± 0.02
Dynamic Baseline	0.69 ± 0.02	0.23 ± 0.02	0.11 ± 0.01	0.06 ± 0.03
Static Logistic	0.67 ± 0.02	0.22 ± 0.02	0.11 ± 0.01	0.04 ± 0.02
Dynamic Logistic	0.69 ± 0.02	0.25 ± 0.02	0.11 ± 0.01	0.04 ± 0.03
Static DeepMTP	0.66 ± 0.03	0.21 ± 0.04	0.12 ± 0.02	0.24 ± 0.08
Dynamic DeepMTP	0.67 ± 0.03	0.22 ± 0.04	0.12 ± 0.01	0.24 ± 0.09
Static FactorizationMachine	0.67 ± 0.02	0.22 ± 0.02	0.11 ± 0.01	0.13 ± 0.02
Dynamic FactorizationMachine	0.69 ± 0.02	0.24 ± 0.03	0.11 ± 0.01	0.15 ± 0.05
