# Supplementary material for: Machine-learning-based prediction of disability progression in multiple sclerosis: An observational, international, multi-center study
Source: PLOS Digit Health. 2024 Jul 25;3(7):e0000533. doi: 10.1371/journal.pdig.0000533 (PMC11271865; doi:10.1371/journal.pdig.0000533)
Supplement: S4 Table — ROC-AUC, AUC-PR, Brier Score and ECE of all models on the different MS course subgroups (averages ± standard deviations). Primary Progressive (PP), Relapsing Remitting (RR) and Secondary Progressive are considered (SP). Cohort of patients with a least 3 visits with EDSS in the last 3.25 years. (PDF) [file pdig.0000533.s009.pdf]

Model	MSCourse	ROC-AUC	AUC-PR	Brier	ECE
Attention	PP	0.65 ± 0.01	0.33 ± 0.04	0.16 ± 0.01	0.07 ± 0.02
Attention	RR	0.7 ± 0.0	0.21 ± 0.01	0.09 ± 0.0	0.06 ± 0.03
Attention	SP	0.65 ± 0.01	0.33 ± 0.03	0.17 ± 0.01	0.1 ± 0.05
RNN	PP	0.65 ± 0.02	0.33 ± 0.05	0.16 ± 0.01	0.05 ± 0.02
RNN	RR	0.69 ± 0.0	0.19 ± 0.01	0.09 ± 0.0	0.05 ± 0.02
RNN	SP	0.64 ± 0.01	0.31 ± 0.02	0.17 ± 0.01	0.05 ± 0.02
Static Bayesian NN	PP	0.62 ± 0.01	0.29 ± 0.04	0.16 ± 0.01	0.12 ± 0.07
Static Bayesian NN	RR	0.64 ± 0.01	0.16 ± 0.01	0.09 ± 0.01	0.09 ± 0.05
Static Bayesian NN	SP	0.62 ± 0.02	0.29 ± 0.02	0.17 ± 0.01	0.07 ± 0.02
Dynamic Bayesian NN	PP	0.66 ± 0.01	0.34 ± 0.03	0.16 ± 0.01	0.09 ± 0.05
Dynamic Bayesian NN	RR	0.7 ± 0.01	0.2 ± 0.0	0.09 ± 0.01	0.09 ± 0.03
Dynamic Bayesian NN	SP	0.64 ± 0.01	0.32 ± 0.02	0.17 ± 0.01	0.11 ± 0.02
Static Baseline	PP	0.62 ± 0.03	0.28 ± 0.05	0.17 ± 0.01	0.1 ± 0.04
Static Baseline	RR	0.64 ± 0.01	0.16 ± 0.01	0.09 ± 0.01	0.06 ± 0.04
Static Baseline	SP	0.62 ± 0.01	0.29 ± 0.02	0.17 ± 0.01	0.07 ± 0.04
Dynamic Baseline	PP	0.63 ± 0.03	0.32 ± 0.05	0.16 ± 0.01	0.09 ± 0.03
Dynamic Baseline	RR	0.69 ± 0.01	0.19 ± 0.0	0.09 ± 0.01	0.05 ± 0.02
Dynamic Baseline	SP	0.63 ± 0.01	0.31 ± 0.02	0.17 ± 0.01	0.1 ± 0.04
Static Logistic	PP	0.59 ± 0.03	0.27 ± 0.05	0.17 ± 0.01	0.1 ± 0.05
Static Logistic	RR	0.64 ± 0.01	0.15 ± 0.01	0.09 ± 0.01	0.07 ± 0.01
Static Logistic	SP	0.59 ± 0.01	0.27 ± 0.03	0.17 ± 0.01	0.04 ± 0.01
Dynamic Logistic	PP	0.63 ± 0.01	0.31 ± 0.03	0.16 ± 0.01	0.09 ± 0.03
Dynamic Logistic	RR	0.68 ± 0.01	0.19 ± 0.01	0.09 ± 0.01	0.05 ± 0.01
Dynamic Logistic	SP	0.61 ± 0.01	0.3 ± 0.04	0.17 ± 0.01	0.08 ± 0.02
Static DeepMTP	PP	0.62 ± 0.04	0.3 ± 0.05	0.17 ± 0.01	0.21 ± 0.06
Static DeepMTP	RR	0.63 ± 0.02	0.15 ± 0.01	0.09 ± 0.01	0.25 ± 0.11
Static DeepMTP	SP	0.61 ± 0.02	0.3 ± 0.03	0.17 ± 0.01	0.21 ± 0.08
Dynamic DeepMTP	PP	0.62 ± 0.04	0.31 ± 0.06	0.17 ± 0.01	0.19 ± 0.05
Dynamic DeepMTP	RR	0.66 ± 0.02	0.18 ± 0.01	0.09 ± 0.01	0.18 ± 0.05
Dynamic DeepMTP	SP	0.62 ± 0.02	0.31 ± 0.03	0.17 ± 0.01	0.18 ± 0.05
Static FactorizationMachine	PP	0.62 ± 0.02	0.29 ± 0.04	0.17 ± 0.01	0.19 ± 0.06
Static FactorizationMachine	RR	0.64 ± 0.01	0.16 ± 0.01	0.09 ± 0.01	0.24 ± 0.07
Static FactorizationMachine	SP	0.63 ± 0.01	0.3 ± 0.03	0.17 ± 0.01	0.15 ± 0.05
Dynamic FactorizationMachine	PP	0.64 ± 0.02	0.31 ± 0.03	0.17 ± 0.01	0.16 ± 0.04
Dynamic FactorizationMachine	RR	0.68 ± 0.0	0.19 ± 0.01	0.09 ± 0.01	0.2 ± 0.01
Dynamic FactorizationMachine	SP	0.63 ± 0.01	0.31 ± 0.02	0.17 ± 0.01	0.18 ± 0.02
