# Supplementary material for: Machine-learning-based prediction of disability progression in multiple sclerosis: An observational, international, multi-center study
Source: PLOS Digit Health. 2024 Jul 25;3(7):e0000533. doi: 10.1371/journal.pdig.0000533 (PMC11271865; doi:10.1371/journal.pdig.0000533)
Supplement: S6 Table — ROC-AUC, AUC-PR, Brier Score and ECE by severity subgroup (averages ± standard deviations). Low severity patients are defined as the ones with EDSS ≤ 5.5 at baseline, while high severity patients are defined as having EDSS > 5.5 at baseline. Cohort of patients with a least 3 visits with EDSS in the last 3.25 years. (PDF) [file pdig.0000533.s011.pdf]

Model	EDSS	ROC-AUC	AUC-PR	Brier	ECE
Attention	$EDSS \leq 5.5$	0.72 ± 0.01	0.26 ± 0.01	0.09 ± 0.0	0.07 ± 0.04
Attention	$EDSS > 5.5$	0.65 ± 0.01	0.27 ± 0.04	0.15 ± 0.01	0.07 ± 0.02
RNN	$EDSS \leq 5.5$	0.71 ± 0.01	0.24 ± 0.02	0.1 ± 0.0	0.04 ± 0.03
RNN	$EDSS > 5.5$	0.64 ± 0.01	0.26 ± 0.03	0.15 ± 0.02	0.05 ± 0.03
Static Bayesian NN	$EDSS \leq 5.5$	0.67 ± 0.01	0.21 ± 0.01	0.1 ± 0.0	0.1 ± 0.04
Static Bayesian NN	$EDSS > 5.5$	0.63 ± 0.01	0.25 ± 0.03	0.15 ± 0.02	0.09 ± 0.04
Dynamic Bayesian NN	$EDSS \leq 5.5$	0.72 ± 0.01	0.25 ± 0.01	0.09 ± 0.0	0.08 ± 0.04
Dynamic Bayesian NN	$EDSS > 5.5$	0.64 ± 0.02	0.26 ± 0.03	0.15 ± 0.01	0.11 ± 0.03
Static Baseline	$EDSS \leq 5.5$	0.67 ± 0.01	0.2 ± 0.02	0.1 ± 0.01	0.07 ± 0.05
Static Baseline	$EDSS > 5.5$	0.63 ± 0.01	0.25 ± 0.03	0.15 ± 0.02	0.09 ± 0.04
Dynamic Baseline	$EDSS \leq 5.5$	0.71 ± 0.01	0.24 ± 0.01	0.1 ± 0.01	0.09 ± 0.03
Dynamic Baseline	$EDSS > 5.5$	0.63 ± 0.01	0.26 ± 0.04	0.15 ± 0.02	0.09 ± 0.03
Static Logistic	$EDSS \leq 5.5$	0.66 ± 0.01	0.2 ± 0.01	0.1 ± 0.01	0.03 ± 0.01
Static Logistic	$EDSS > 5.5$	0.61 ± 0.02	0.24 ± 0.04	0.15 ± 0.01	0.05 ± 0.01
Dynamic Logistic	$EDSS \leq 5.5$	0.7 ± 0.0	0.24 ± 0.02	0.1 ± 0.0	0.06 ± 0.04
Dynamic Logistic	$EDSS > 5.5$	0.62 ± 0.01	0.25 ± 0.04	0.15 ± 0.01	0.07 ± 0.03
Static DeepMTP	$EDSS \leq 5.5$	0.65 ± 0.02	0.19 ± 0.02	0.1 ± 0.0	0.23 ± 0.06
Static DeepMTP	$EDSS > 5.5$	0.63 ± 0.02	0.25 ± 0.04	0.15 ± 0.02	0.21 ± 0.05
Dynamic DeepMTP	$EDSS \leq 5.5$	0.69 ± 0.02	0.22 ± 0.02	0.1 ± 0.0	0.18 ± 0.03
Dynamic DeepMTP	$EDSS > 5.5$	0.62 ± 0.02	0.26 ± 0.04	0.15 ± 0.02	0.19 ± 0.08
Static FactorizationMachine	$EDSS \leq 5.5$	0.67 ± 0.01	0.21 ± 0.02	0.1 ± 0.0	0.18 ± 0.06
Static FactorizationMachine	$EDSS > 5.5$	0.63 ± 0.01	0.26 ± 0.04	0.15 ± 0.02	0.18 ± 0.06
Dynamic FactorizationMachine	$EDSS \leq 5.5$	0.7 ± 0.01	0.23 ± 0.01	0.1 ± 0.0	0.14 ± 0.03
Dynamic FactorizationMachine	$EDSS > 5.5$	0.64 ± 0.01	0.26 ± 0.03	0.15 ± 0.02	0.22 ± 0.05
