# Supplementary material for: Machine-learning-based prediction of disability progression in multiple sclerosis: An observational, international, multi-center study
Source: PLOS Digit Health. 2024 Jul 25;3(7):e0000533. doi: 10.1371/journal.pdig.0000533 (PMC11271865; doi:10.1371/journal.pdig.0000533)
Supplement: S8 Table — Features are ranked by order of importance for the Dynamic Model. Feature importance is assessed by the average difference in performance when the specific feature is shuffled. Averages ± standard deviations are reported. (PDF) [file pdig.0000533.s013.pdf]

Covariate	ROC-AUC	AUC-PR	Brier	ECE
EDSS at 0	$0{\cdot}04 \pm 0{\cdot}0$	$0{\cdot}04 \pm 0{\cdot}0$	$-0{\cdot}0 \pm 0{\cdot}0$	$0{\cdot}07 \pm 0{\cdot}03$
Date reference	$0{\cdot}04 \pm 0{\cdot}01$	$0{\cdot}02 \pm 0{\cdot}01$	$-0{\cdot}0 \pm 0{\cdot}0$	$-0{\cdot}01 \pm 0{\cdot}03$
Mean EDSS last3y	$0{\cdot}02 \pm 0{\cdot}01$	$0{\cdot}02 \pm 0{\cdot}01$	$-0{\cdot}0 \pm 0{\cdot}0$	$0{\cdot}01 \pm 0{\cdot}03$
Mean KFS 1 last3y	$0{\cdot}01 \pm 0{\cdot}0$	$0{\cdot}01 \pm 0{\cdot}01$	$-0{\cdot}0 \pm 0{\cdot}0$	$0{\cdot}02 \pm 0{\cdot}05$
Onset date reference	$0{\cdot}01 \pm 0{\cdot}01$	$0{\cdot}01 \pm 0{\cdot}0$	$-0{\cdot}0 \pm 0{\cdot}0$	$0{\cdot}02 \pm 0{\cdot}03$
MSCOURSE AT VISIT SP	$0{\cdot}01 \pm 0{\cdot}0$	$0{\cdot}01 \pm 0{\cdot}0$	$-0{\cdot}0 \pm 0{\cdot}0$	$0{\cdot}01 \pm 0{\cdot}02$
Mean KFS 2 last3y	$0{\cdot}01 \pm 0{\cdot}0$	$0{\cdot}01 \pm 0{\cdot}0$	$-0{\cdot}0 \pm 0{\cdot}0$	$0{\cdot}02 \pm 0{\cdot}04$
Disease duration at 0 years	$0{\cdot}01 \pm 0{\cdot}0$	$0{\cdot}01 \pm 0{\cdot}0$	$-0{\cdot}0 \pm 0{\cdot}0$	$-0{\cdot}01 \pm 0{\cdot}03$
Std EDSS last3y	$0{\cdot}01 \pm 0{\cdot}0$	$0{\cdot}01 \pm 0{\cdot}0$	$-0{\cdot}0 \pm 0{\cdot}0$	$-0{\cdot}01 \pm 0{\cdot}02$
Mean KFS AMBULATION last3y	$0{\cdot}01 \pm 0{\cdot}01$	$0{\cdot}0 \pm 0{\cdot}0$	$-0{\cdot}0 \pm 0{\cdot}0$	$0{\cdot}01 \pm 0{\cdot}02$
Others	$< 0{\cdot}01$	$< 0{\cdot}01$	$< 0{\cdot}01$	$< 0{\cdot}01$
