# Supplementary material for: Machine-learning-based prediction of disability progression in multiple sclerosis: An observational, international, multi-center study
Source: PLOS Digit Health. 2024 Jul 25;3(7):e0000533. doi: 10.1371/journal.pdig.0000533 (PMC11271865; doi:10.1371/journal.pdig.0000533)
Supplement: S9 Table — List of hyperparameters used for training the models. (PDF) [file pdig.0000533.s014.pdf]

Model: Temporal Attention	
Epochs	100
Dropout	[0.3,0.4,0.5,0.6,0.7,0.8]
Learning rate	0.0001
Hidden dimension	128
Batch size	1024
Layers	2
