# Supplementary material for: Machine-learning-based prediction of disability progression in multiple sclerosis: An observational, international, multi-center study
Source: PLOS Digit Health. 2024 Jul 25;3(7):e0000533. doi: 10.1371/journal.pdig.0000533 (PMC11271865; doi:10.1371/journal.pdig.0000533)
Supplement: S10 Table — List of hyperparameters used for training the models. (PDF) [file pdig.0000533.s015.pdf]

Model: Multi-Layer Perceptron	
Epochs	100
Dropout	[0.,0.1,0.2,0.3,0.4,0.5]
Learning rate	0.001
Hidden dimension	128
Batch size	1024
Layers	5
