# Supplementary material for: Machine-learning-based prediction of disability progression in multiple sclerosis: An observational, international, multi-center study
Source: PLOS Digit Health. 2024 Jul 25;3(7):e0000533. doi: 10.1371/journal.pdig.0000533 (PMC11271865; doi:10.1371/journal.pdig.0000533)
Supplement: S13 Table — List of hyperparameters used for training the models. (PDF) [file pdig.0000533.s018.pdf]

Model: Factorizing Machines	
Epochs	20
Learning rate	0.0001
Hidden dimension	[3, 5, 7, 9, 11, 13, 15]
