# Supplementary material for: Machine-learning-based prediction of disability progression in multiple sclerosis: An observational, international, multi-center study
Source: PLOS Digit Health. 2024 Jul 25;3(7):e0000533. doi: 10.1371/journal.pdig.0000533 (PMC11271865; doi:10.1371/journal.pdig.0000533)
Supplement: S1 Text — Description of the Bayesian neural networks, DeepMTP, and Factorization Machines models. (PDF) [file pdig.0000533.s020.pdf]

Models description

Bayesian Neural Networks

Introduced by Gal et al. [1], Monte Carlo Dropout is an approximate ensemble method for Bayesian Neural Networks (Bayesian NN or BNN). They prove that a Neural Network with dropout layers and L2-regularization approximates the predictive posterior distribution of a Gaussian process for a given data set. The resulting ensemble is in general well-calibrated [2], and more accurate than its non-Bayesian counterpart due to the regularizing effect. The Bayesian NN implemented in this work is identical to the baseline neural network previously introduced, with a few key differences. First, dropout [3] is applied between every layer. Second, the logits of the network are modeled as Gaussian distributions, rather than point estimates. Third, the loss function is a modified cross-entropy loss, that samples from the aforementioned logit distributions [2]. While Kendall et al. [2] define a loss that allows capturing aleatoric uncertainty, we simplify it further to work for our binary classification. We use Monte Carlo integration to approximate the distribution.

The resulting network has a way of expressing epistemic (model-bound) uncertainty by using dropout, creating a distribution over the model weights. On the other hand, the resulting BNN also has a way of expressing aleatoric uncertainty, by modelling the output of the network as a Gaussian distribution in logit space.

We define the network to have two outputs, μ and $\log \sigma^2$. Instead of learning variance, log variance is learnt to constrain the value to be positive. Our loss function for a single input, with T the amount of Monte Carlo integration samples, and y the true label is defined as follows:

$$\hat{p}_t = \mu + \sigma \epsilon_t, \quad \epsilon_t \sim \mathcal{N}(0, I)$$
$$\mathcal{L} = -\log \frac{1}{T} \sum_t \hat{p}_t^{y_i} (1 - \hat{p}_t)^{1-y_i}$$
$$\mathcal{L} = -\log \frac{1}{T} \sum_t \exp\left(y \log \hat{p}_t + (1-y) \log(1 - \hat{p}_t)\right)$$

DeepMTP

The DeepMTP framework was introduced by Iliadis et al. [4] as a unified approach for multi-target prediction (MTP) problems. Applications that fall under the umbrella of MTP are concerned with the simultaneous prediction of multiple target variables. Even though this work focuses on the prediction of a single binary variable (patient progresses or not), we are able to use the DeepMTP framework by applying a multi-task trick. To achieve this, we select one categorical feature from the available data set (country) and create multiple targets (or tasks), thus forming a multi-task learning problem. By doing this, the goal becomes the prediction of the progression of a patient depending on the country (s)he is residing in. Even though the natural benchmark comparison of a multi-task problem is a collection of models that are trained on subsets of the original data set belonging to a single country (single-task models), we believe that this is out of the scope of this work. For this reason, after prediction, we collapse the tasks and create a single prediction that is comparable with the other methods tested in this paper.

In terms of architecture, DeepMTP uses a two-branch architecture that is flexible enough to be adapted for the different MTP prediction settings. In this specific multi-task case, the first branch encodes the same features as all the other methods, and the second uses a one-hot encoded vector that maps to a given country. Both branches are comprised of one or more fully connected layers, and their outputs are combined

using a dot product. The single value resulting from the dot product is the output of the entire network (progression or not).

Factorization Machines

In 2010, Rendle introduced Factorization Machines (FM) as a new model class that combines the advantages of Support Vector Machines (SVM) with factorization models [5]. Factorization machines model interactions between features using factorized parameters. The prediction function of degree two, meaning that pairwise interactions represent the highest degree of interaction considered, is given by:

$$f(\boldsymbol{x}) := w_0 + \sum_{i=1}^{n} w_i x_i + \sum_{i=1}^{n} \sum_{j=i+1}^{n} \langle \boldsymbol{v_i}, \boldsymbol{v_j} \rangle \, x_i x_j \; ,$$

with model parameters $w_0 \in \mathbb{R}$, $\boldsymbol{w} \in \mathbb{R}^n$ and $\boldsymbol{V} \in \mathbb{R}^{n \times k}$, and the dot product $\langle \boldsymbol{v_i}, \boldsymbol{v_j} \rangle := \sum_{f=1}^{k} v_{i,f} v_{j,v}$.

Factorization Machines are more widely applicable than regular factorization models such as matrix factorization, as they naturally include features as well and learn to map them into a lower-dimensional latent factor space. This behaviour explains why FM can be surprisingly successful when working with categorical features (e.g. country, sex), even under high sparsity. Also, thanks to their linear time complexity, they are often applied in large real-world recommendation data sets [6].

In this work, we used stochastic gradient descent with adaptive regularization as a learning method [6, 7]. A Python implementation is available at `https://github.com/godpgf/pylibfm`. The main hyper-parameter to tune is the size k of the latent factor space.

References

1. Gal Y, Ghahramani Z. Dropout as a bayesian approximation: Representing model uncertainty in deep learning. In: international conference on machine learning. PMLR; 2016. p. 1050–1059.

2. Kendall A, Gal Y. What Uncertainties Do We Need in Bayesian Deep Learning for Computer Vision? arXiv:170304977 [cs]. 2017;.

3. Srivastava N, Hinton G, Krizhevsky A, Sutskever I, Salakhutdinov R. Dropout: a simple way to prevent neural networks from overfitting. The journal of machine learning research. 2014;15(1):1929–1958.

4. Iliadis D, De Baets B, Waegeman W. Multi-target prediction for dummies using two-branch neural networks. Machine Learning. 2022;doi:10.1007/s10994-021-06104-5.

5. Rendle S. Factorization machines. In: 2010 IEEE International conference on data mining. IEEE; 2010. p. 995–1000.

6. Rendle S. Learning recommender systems with adaptive regularization. In: Proceedings of the fifth ACM international conference on Web search and data mining; 2012. p. 133–142.

7. Rendle S. Factorization machines with libfm. ACM Transactions on Intelligent Systems and Technology (TIST). 2012;3(3):1–22.
