# Supplementary material for: Machine-learning-based prediction of disability progression in multiple sclerosis: An observational, international, multi-center study
Source: PLOS Digit Health. 2024 Jul 25;3(7):e0000533. doi: 10.1371/journal.pdig.0000533 (PMC11271865; doi:10.1371/journal.pdig.0000533)
Supplement: Attachment — Submitted filename: ResponseToReviewers.pdf [file pdig.0000533.s021.pdf]

Machine-learning-based prediction of disability progression in multiple sclerosis: an observational, international, multi-center study

Authors rebuttal

We first want to thank all reviewers for their constructive feedback that allowed us to significantly improve our paper. We are happy to report that we have taken action for every single comment raised by the reviewers. We summarize the changes in the paper below and direct answers to each reviewer's questions are provided in the subsequent sections.

Overall changes to the manuscript.

- We clarified the motivation for using the confirmed disability progression label as our binary prediction label. This label definition was previously introduced in Kalincik et al. [1] in an attempt to provide an actionable yet clinically meaningful binary label to reflect disability progression. We made the relationship to Kalincik et al. more explicit. We also expanded the discussion to reflect the pros and cons of such a label.
- We have clarified that our models predict a probability of disease progression, and not only a binary label of progression, and nuanced its implication for clinical practice.
- We have improved the discussion regarding our choice of requiring a minimum clinical follow-up period, which excludes newly diagnosed MS patients. This decision was motivated by prior work, which showed that clinical history leads to more accurate prognosis, a finding that we confirm in this study.
- We have significantly reworked the manuscript to improve clarity. In particular, the methods section has been refactored to improve the description of the data preprocessing pipeline (such as the creation of episodes and the confirmed disability progression label). We have also included additional details regarding the training and evaluation of the models.
- According to editorial policies, we have removed the figures from the main text and provided them in separate files. We kept the captions of figures in the text. We also added a list of legends at the end of our supporting information.

[1] Tomas Kalincik, Ali Manouchehrinia, Lukas Sobisek, Vilija Jokubaitis, Tim Spelman, Dana Horakova, Eva Havrdova, Maria Trojano, Guillermo Izquierdo, Alessandra Lugaresi, et al. Towards personalized therapy for multiple sclerosis prediction of individual treatment response. Brain, 140(9):2426–2443, 2017

Direct answers to reviewers comments

REVIEWER 1

Comment : "The authors claim to train models 'for disability progression prediction in MS.' While this may be the end goal of the work, the work presented in this manuscript is a classification of whether a change will occur or not, not a prediction of the actual disease progression. The presented work is an important stepping stone, however, it cannot address the motivation suggests for uses of patient planning and care. Please reframe the paper in a more appropriate manner to reflect the results presented."

Answer: We agree with the reviewer and made clear in the introduction that our prediction target is a binary variable representing the occurrence of a confirmed disability progression event in the next two years. We furthermore clarified that our models predict the *probability* of progression, i.e., a value between 0 and 1 that can be interpreted as the probability of disease progression. We also modified Figure 1 to show that our model outputs the *probability* of progression.

We also added a paragraph in the discussion about the granularity of the classification target and linked it more directly to the confirmed disability progression label proposed by Kalincik et al [1]. Indeed, MS being such a complex disease, the translation to a single definition of disease progression inevitably leads to simplifications. We have also clarified our dependencies on Kalincik et al. for the disability progression label in paragraph 4.3.

Here is an excerpt of the updated discussion section:

"EDSS itself, as an attempt to quantify progression on one-dimensional scale, lacks the expressivity to reliably encode the progression of the disease. What is more, we framed disability progression as a classification task, which is more granular than predicting future EDSS but is more amenable for machine learning. Despite these imperfections, the confirmed disability progression label used in this work has been proven clinically useful, striking a good balance between abstraction and expressivity."

[1] Tomas Kalincik, Ali Manouchehrinia, Lukas Sobisek, Vilija Jokubaitis, Tim Spelman, Dana Horakova, Eva Havrdova, Maria Trojano, Guillermo Izquierdo, Alessandra Lugaresi, et al. Towards personalized therapy for multiple sclerosis prediction of individual treatment response. Brain, 140(9):2426–2443, 2017

Comment : "The exclusion criteria limit those who are newly diagnosed and/or currently have a steady disease state. This is partially addressed as a limitation in the discussion but this needs to be expanded. These exclusions limit the model to predict whether a change will occur in those who already present changes instead of PwMS overall."

Answer: We have expanded our discussion of this important limitation of our work. In particular, we argued that

"This [criterion] limits the application of our model to patients with an already established clinical MS history. This decision was motivated by prior work, which showed that clinical history leads to more accurate prognosis, a finding that we confirm in this study. A new dedicated model would be required for disability progression in patients with shorter clinical history. Nevertheless, MS being a chronic disease, many patients would still satisfy the 3 visits follow-up inclusion criteria (64% in the MSBase cohort)."

Furthermore, we would like to note that:

- Since pwMS at the start of their disease have no measured longitudinal variables, this would require a different modeling strategy.
- Since MS is a chronic disease, almost all pwMS have the disease for more than a few years, which makes this model applicable to most pwMS.

Comment: "P.18 is this definition of disease progression a clinical standard or is it introduced for this work? Need to define and motivate or cite."

Answer: The definition of confirmed disability progression used in our work comes from Kalincik et al. [1] and was designed to translate the clinical concept of disability progression into a label amenable to statistical and machine learning models application.

We have also clarified our dependencies on Kalincik et al. for the disability progression label in paragraph 4.3 of the methods section.

[1] Tomas Kalincik, Ali Manouchehrinia, Lukas Sobisek, Vilija Jokubaitis, Tim Spelman, Dana Horakova, Eva Havrdova, Maria Trojano, Guillermo Izquierdo, Alessandra Lugaresi, et al. Towards personalized therapy for multiple sclerosis prediction of individual treatment response. Brain, 140(9):2426–2443, 2017

Comment: " Please provide more details on model training and evaluation (eg. training epochs)"

Answer: We thank the reviewer for this suggestion. For each model in our comparisons, we have now included a table displaying the main hyperparameters of each method and the values that were used in the experiment. For clarity we have kept these tables in the Supplementary (F.4.) and refer to them in the Methods section.

Comment: "Several models are presented with very similar results and claims are made about which is best. Please add significance tests to determine if these differences are significant."

Answer: We have added p-values from unpaired t-test in Table 2.

Comment: "Throughout the manuscript there is a mix of present and past tense"

Answer: We now use past tense for describing our work over the whole manuscript. We kept the present tense in sentences that directly relate to the organization of the manuscript, such as "The results are presented in Figure X".

Comment: "Define all acronyms before they are used in text"

Answer: We have checked the occurrence of all acronyms in the text and have made sure to provide a definition at the first occurence.We have now defined all acronyms.

Comment: "Need to include definitions of acronyms in table captions"

Answer: We have revised our captions (Tables and Figures) to ensure that acronyms were properly defined, thereby improving the clarity of our manuscript.

Comment: "Define all elements of equations clearly (e.g. page 26; Parameter p seems to be defined in figured but not text)"

Answer: All elements of the equations have now been clearly defined. p(.) in the caption of Figure 7 stands for the *probability*. We have edited the caption to make this clearer.

Comment: "Some results, such as table 5, may be easier to interpret as a figure/bar chart"

Answer: This is a great idea. We are pleased to report that we have transformed Table 5 into Figure 4, where the features importance are now visualized as a bar plot. For completeness, we have kept the original table in the Supplementary Materials (Table S7).

Comment: "Please fix reference to supplementary materials on page 24 line 329 ?? and the corresponding table in supplementary materials."

Answer: We thank the reviewer for catching this mistake. We have fixed it.

REVIEWER 2

Comment: "The introduction stays vague in developing the rationale for the parameters of interest. Also, no hypotheses are formulated. Instead, the results are already summarized which feels like a repetition of the abstract."

Answer: We added the following text in the introduction:

"In this article, we aimed to develop a model with three specific goals. Firstly, it should predict the *probability* (a value between 0 and 1) of disability progression for a PwMS within the next two years, instead of just a binary target (0 or 1, i.e., disease progression or no disease progression). Secondly, it should be applicable to a well-defined, relevant, and large patient population. Thirdly, all variables used in the model should be available in routine clinical care. A successful combination of these three goals allows one to employ this model in a clinical impact study, and presents a significant step towards clinical applicability."

We hope this clarifies the rationale of the developed model, and the contribution of the paper to the literature.

Comment: "The Cohort section of the results is mainly methods, I would therefore suggest removing this information."

Answer: We warmly welcome this suggestion and have significantly shortened the cohort description in the results section and expanded our exposition of the cohort definition in the methods section (Section 4.2).

Comment: "please explain all the abbreviations. Also the captions for the tables are usually above the table, if not requested differently."

Answer: We have checked the occurrence of acronyms in all Tables and have defined them in the captions. We have also moved the caption above all tables.

Comment: "Was the model also tested on less input span? Would be interesting what the minimal number of required monitoring points would be."

Answer: The model was not tested on shorter observation windows. In the discussion, we expanded on this important limitation of our work (also at the request of reviewer 1).
To reiterate our comments made to reviewer 1:

- Since pwMS at the start of their disease have no measured longitudinal variables, this would require different modeling.
- Since MS is a chronic disease, almost all pwMS have the disease for more than a few years, which makes this model applicable to most pwMS.

Comment: "The discussion has not a single reference to other published models (that were named in the introduction). Please report and compare the results to existing prediction models to give the reader a fair chance to evaluate the reported outcome."

Answer: We added a paragraph in the discussion on how the performance of our model compares to previous works.

Comment: "Although I appreciate a detailed description of the methods, the amount of information is just overwhelming. Describing why a parameter is abbreviated is too much (e.g. why w is chosen to abbreviate worsening). The methods are too narrative and would be shorter with less storytelling. Also, the methods are quite chaotic."

Answer: We have refactored the methods section to improve the clarity. In particular, we edited the cohort, episodes, and labels subsection extensively and made sure to stay comprehensive while being more direct and clear in our explanations. We have moved the detailed descriptions of the different models to the supplements.

Comment: "If the section about valid/non-valid samples would be clearer, Figure 5 would not be needed."

Answer: We thank the reviewer for this feedback. We clarified the criteria for validity of clinical episodes in Section 4.4. In particular, we report that episodes were considered valid if they met the following criteria:

- A valid confirmed disability progression label (wc) could be computed at t = 0, as defined in Section 4.3.
- The time at which the predictions were made were after 1990 (t0 > 1990, Jan 1st). This ensured that we had a cohort of patients from decades where disease modifying therapies (DMTs) were available [26].
- There were at least k EDSS measurement in the last last three years and three months of the observation window, where k is either three or six measurements

We hope this greatly clarifies the definition of episodes. For sake of completeness and clarity, we kept Figure 6, as we believe it can help the readers understand our construction / data processing.

Comment: "Line 240 ff: why were the two cohorts defined? There is so much description of things that are not important, but then this is not clear."

Answer: We agree with the reviewer that the two cohorts are confusing. We defined two cohorts to study the impact of follow-up on the performance of the models, which has been clarified in Section 4.4. Our results focus on the cohort with a minimum of three EDSS measurements, and we have thus removed the discussion of the six EDSS cohorts in the main text. We nevertheless refer the reader to the supplements for results on the six EDSS cohorts. We did not notice a significant difference in performance between both cohorts.

Comment: "What was the rationale behind training the model with these long time spans? For efficient patient management, much shorter time frames are required. Also, which real predictive value has the model, if so much input is needed?"

Answer: This decision was motivated by prior work, which showed that clinical history leads to more accurate prognosis, a finding that we confirm in this study. A new dedicated model would be required for disability progression in patients with shorter clinical history. Nevertheless, MS being a chronic disease, most patients would still satisfy the 3 visits follow-up inclusion criteria (64% in the MSBase cohort).

Comment: "Line 291: 'If data can be missing...' probably better to use 'If data was missing...'"

Answer: Here we mean that some features can have missing information. In this case, a category unknown is created. We rephrased it to "If that feature contains missing occurrences, the category "unknown" is added." for clarity.

Comment: "Line 330: supplementary material number is not valid."

Answer: Thank you for spotting this mistake. We have now corrected it.

Comment: "It is completely unclear to me why first 3 different models are named and described briefly, followed by a section where they are again explained in great detail but differently named and in a different order then the summary description. any reader that is not deeply informed about machine learning will be lost. I encourage the authors to be concise and precise."

Answer: We have refactored the methods section to improve clarity.

REVIEWER 3

Comment: "The authors claim to evaluate the performance of their model on an external dataset (where the "externalization" refers to splitting the data by patient center). Have the authors conducted any data-distribution level visualizations/statistics to verify whether the data occupy different distributions? Specifically, have the authors considered other axes of data heterogeneity in addition to patient population?"

Answer: We warmly welcome this suggestion. Our external validation strategy aims at stress testing the models in an environment similar to the deployment to a new clinic which was not part of MSBase.

Characterizing the discrepancy between high dimensional distributions is difficult. Nevertheless, we attempted to characterize the heterogeneity of the different centers by creating embeddings built from earth mover distance between centers. These two-dimensional embeddings for each center in the dataset are displayed in Figure S.3. They were obtained by representing each center as the set of vectors of static variables for each episode and computing a distance between centers using earth mover distance. The two-dimensional visualization was obtained by using multidimensional scaling (MDS). We observed that centers from the same country tend to cluster together, suggesting inter-country variability. This supports our external validation strategy that leaves whole centers in the test set.

Comment: "It is great that the authors assessed the degree of model calibration, together with the Brier score, in their work. Given the size of the dataset, have the authors considered the degree to which model predictions are repeatable across patients and/or explainable?"

Answer: The models are repeatable in the sense that the same input always gives the same output. We have not tested repeatability in the sense of doing the same measurements one day apart and quantifying the variation of the predictions (see, e.g., "Improving the repeatability of deep learning models with Monte Carlo dropout, A. Lemay et al., npj digital medicine, 2022"). Such a study would require a significant effort, since such measurements are not currently available in the MSBase dataset.
