# Supplementary material for: Machine-learning-based prediction of disability progression in multiple sclerosis: An observational, international, multi-center study
Source: PLOS Digit Health. 2024 Jul 25;3(7):e0000533. doi: 10.1371/journal.pdig.0000533 (PMC11271865; doi:10.1371/journal.pdig.0000533)
Supplement: Attachment — Submitted filename: MS_rebuttal.pdf [file pdig.0000533.s022.pdf]

Machine-learning-based prediction of disability progression in multiple sclerosis: an observational, international, multi-center study

Dear Editor and Reviewers,

Thank you for your invaluable feedback and support. We are glad to read that our previous clarifications and answers were greatly appreciated and that you recognize the importance of our work. Your collective feedback grealty contributed to making our work stronger and our manuscript more accurate and rigorous.

We are happy to report that we now have implemented the further suggestions you provided for our manuscript. Please find our detailed answers to each point you raised below. The attached manuscript also contains track-changes with respect to the original version.

We hope you will appreciate our efforts in answering your latest comments.

Thank you for your support,

Best regards,

The authors

Reviewer #1

"Thank you for addressing the comments. The additional details and clarifications are greatly appreciated. Based on the p-values added in table 2, there is not sufficient evidence that the attention model performs best. Please adjust the wording throughout (including introduction) to address this. Additionally, there are a few formatting (eg. p7 line ~52 line break) and wording errors that could use one more readthrough."

Thank you for your encouragement. We are pleased to report that we have now revised the manuscript to reflect the lack of statistical significance in the difference of performance of the models. In particular, we have removed the emphasis on the temporal attention model in the introduction and the abstract. We also revised the results and discussion section to refer to the performance of the different models, rather than the temporal transformer only.

Reviewer #4

"This manuscript presents a machine learning approach for predicting if a person with multiple sclerosis will experience disability progression in the next two years based on data commonly collected during routine clinical care. Models were trained on a very large, multi-national dataset (MSBase). I was not a reviewer on the prior version of the manuscript, but I have reviewed the comments from prior reviewers and believe that the authors have adequately addressed many of their concerns. In my review, I have identified several areas (below) that would benefit from revision to strengthen this manuscript and further highlight this important work."

- "Abstract, Methods: typo – expended -> expanded"

Thank you for spotting this typo. We have fixed it.

- "line64: What is the scientific or clinical rationale for predicting progression in the next two years? Why not 1 year? Or 6 months? This seems to have also been a question from the prior reviewers that has not been addressed and would be helpful for readers."

The chosen time window of two years is between short and long timescales, to make sure that enough confirmed progressions will have been observed (so we have a reasonable number of positive labels for training), while keeping the number of patients in the dataset large enough. For short timescales, *e.g.* 6 months, there will be a lot less confirmed progressions, and the training targets will become very imbalanced. For long timescales, e.g. 10 years, one can only use patients with that much follow-up, which would significantly decrease the number of patients we can use for training the models.

Two years is also a clinically sensible time frame during which the disease can progress. There is no clinical or methodological reason why we take 2 years instead of, e.g,. 3 years or 1.5 years. But 2 years is a typical choice in academic literature.

We clarified this choice by adding the following paragraph in the methods section:

"We chose a time horizon of two years as a trade-off between short and long disease time scales. A short horizon would lead to very few confirmed progression in the cohort, making predictive modeling difficult. A long horizon would result in less patients satisfying the inclusion criteria, reducing the sample size. It is a typical choice in the literature and is a relevant timescale for people with multiple sclerosis to plan their lives."

- "lines 114-115: it is noted that the lower performance observed in the primary progressive and secondary progressive subgroups was due to small sample size, but how is that known? Was this tested in some way?"

We agree with the reviewer that our intention may not be reflected in our wording in this paragraph. In this section, we rather mean that we *conjecture* that the low sample size in different subgroups may be

a factor for the decreased performance. Asserting this with confidence would require further experimentation. To clarify our position, we have now revised this paragraph.

"We observed a decreased discrimination performance in the progressive and secondary progressive groups. We conjecture that this is due to the low sample size in these groups."

- "tables 2-4: it would be helpful to note, in the caption, what is being reported after the +/- in these tables."

We have now specified that we report "averages +- standard deviations computed over the different folds" in the caption of all tables (including in the Supplementary Material).

- "tables 3-4: an indication of the training set size for each model being compared would be helpful as it is not exactly clear with all of the subgroups."

We appreciate this suggestion and we have added the size of the training set for the different subgroups in the captions of Tables 3 and 4. We have also added this information in Table 1 with the summary statistics of the cohort, where we wrote the percentage of the clinical episodes with a given EDSS and disease course. We note that most clinical episodes have an EDSS at t=0 equal or lower than 5.5 (83.9%). Most clinical episodes are also categorized in the relapsing-remitting disease course (83.5%). This motivates our conjecture that the low sample size in this subgroup leads to poorer performance (cfr. our answer above).

- "lines 153-154: While it is argued that the presented models are a 'significant advance towards deploying AI in clinical practice in MS' - how do we know what good enough performance is in this context? Is a ROC AUC of 0.72 and PR-AUC of 0.26 sufficient for predicting MS progression? What are the negative byproducts of an incorrect prediction (both false positive and negative)? It would be helpful for readers if these results could be further evaluated and placed in their intended context of use in the discussion section."

First, we want to highlight that the information provided to the patients would be the probability of progression, not a binary label, as pointed out in the third paragraph of the introduction. As such, ROC-AUC and PR-AUC only provide a partial assessment of the performance of the models. The calibration (ECE and Brier Score) is a crucial metric for evaluating the reliability of the predicted probability.

Regarding "the significant advance towards deploying AI in clinical practice", we don't specifically refer to the mere performance of the models here, but rather to the training and evaluation benchmark that was used in our study. Indeed, much care has been given to reproducibility and meaningful clinical evaluation (e.g. carefully following the TRIPOD guidelines). As such, we claim that our model evaluation is as good as one can do before performing a real-world trial. This is also reflected in the objectives of the work laid out in the third paragraph of the introduction.

Nevertheless, we acknowledge that the reported classification performance indicates how useful such a model could be in practice. Here, we note that disability progression is highly stochastic, and predicting it with only routinely collected clinical variables is highly challenging. Our results show that one can extract some signal from the data (and are inline with previously reported measures). The

good calibration also shows that the predicted probabilities are highly reliable, which can be useful for patients and clinicians. For precisely assessing the clinical significance of such models, an highly useful baseline would be to run a study with clinicians and evaluate their performance on the same task. This is a very interesting avenue for future work but that would require significant additional resources.

The negative byproducts of an incorrect prediction are to be understood within the scope of probabilities. Two cases can then occur: overestimation of the probability of progression, or underestimation of the probability of progression. In the former case, this can lead to unnecessary emotional stress to the patient. In the latter case, this can lead to deceptive optimism. If this model is also used to inform treatment decisions, it can lead to the unnecessary use of more potent therapies with additional side effects, or to delaying treatment, losing time to the progression of the disease. Another interesting case study would be to assess to what extent patients would appreciate having a probability of progression, or if they would rather not have this information.

We have added the following text in our discussion to reflect these points:

About what makes the models ready for clinical evaluation:
l.163 "Combined with our rigorous benchmarking, external validation, and our strict adherence to the TRIPOD guidelines, this points towards a readiness of these models to be tested in a clinical impact study."

About what such study would entail, and how to measure the clinical impact of these models:
l.166 "Such study would evaluate the performance of these models in real-world clinical practice, compare them with the predictions of clinicians, and assess the value of such a prediction for pwMS".

About the negative byproducts of inaccurate predictions:
l.168 "Over- or under-prediction of the probability of progression can lead to unnecessary emotional stress or optimism."

- "Discussion: this section would be strengthened with a more detailed discussion of the reported results. For example, the results of Tables 3 and 4 are clearly important, but are not discussed. It would be helpful for the authors to further elucidate what may be causing the observed differences in model performance and to highlight what those performance differences may imply for the translation of this approach into the clinical environment."

We thank the reviewer for this suggestion to improve our manuscript. On top of the changes we made in the results section (*cfr.* our answers above), we have also added a new paragraph in our discussion to reflect on this analysis more directly. The excerpt, at line 197, is presented below.

"Third, our analysis showed that the performance of the different models varied across different patient subgroups. When segmenting the cohort by disease course or by baseline EDSS, we found that the majority subgroup (i.e. relapsing-remitting and $EDSS\_t=0 < 5.5$) showed a better discrimination performance than subgroups with lower prevalence. We conjecture that this difference of performance is due to the lower sample size in the minority subgroups. This finding suggests a more limited value

of the models for pwMS belonging in the minority subgroups. Nevertheless, the difference in calibration was not significantly different."

- "there seems to be an extra (2) between lines 271 and 272."

Thank you for spotting this. We have fixed this mistake.

- "line323: 11.64% progression events suggests a pretty significant imbalance in the data, but it is not clear how this was dealt with in training and evaluating the models. This should be described more clearly."

Using standard practice for unbalanced data, we reweighted each sample proportionally to its label occurrence. We added "We addressed this imbalance by re-weighing each sample proportionally to its label occurrence." in section 4.4. to make this clear.
